# Effects of *IGF1* Knockdown and Overexpression on the Phenotype and Function of Equine Primary Skeletal Muscle Cells

**DOI:** 10.3390/biology15151225

**Published:** 2026-07-23

**Authors:** Yi Su, Wanlu Ren, Yaqi Zeng, Jun Meng, Xinkui Yao, Jianwen Wang

**Affiliations:** 1 College and of Animal Science, Xinjiang Agricultural University, Urumqi 830052, China; 13335339131@163.com (Y.S.); renwanlu@xjau.edu.cn (W.R.); zengyaqi@xjau.edu.cn (Y.Z.); junm86@xjau.edu.cn (J.M.); yaoxinkui@xjau.edu.cn (X.Y.); 2 Xinjiang Key Laboratory of Equine Breeding and Exercise Physiology, Xinjiang Agricultural University, Urumqi 830052, China

**Keywords:** *IGF1*, equine primary skeletal muscle cells, PI3K/Akt signaling pathway, cell proliferation, cell migration, Yili horse

## Abstract

Skeletal muscle serves as the direct executor of movement, and the proliferation and migration capacities of its constituent cells represent the key physiological foundation determining equine endurance and speed. Previous whole-transcriptome analysis has shown that Insulin-like Growth Factor 1 (*IGF1*) is significantly upregulated in *Yili horses* following racing and is involved in the PI3K-Akt signaling pathway; however, its specific regulatory mechanism in equine skeletal muscle cells remains unclear. This study utilized primary equine skeletal muscle cells as a model. By constructing an *IGF1* overexpression plasmid and screening efficient siRNA interference sequences, we employed RT-qPCR and Western Blot techniques to verify gene expression and the activation level of the PI3K/Akt pathway. Cell Counting Kit-8 (CCK-8) assays were used to detect cell proliferation viability, and cell scratch assays were conducted to evaluate migration capacity, aiming to clarify the regulatory effect of *IGF1* on the phenotype and function of equine skeletal muscle cells. Functional experiments demonstrated that *IGF1* overexpression significantly promoted the proliferation viability (*p* < 0.0001) and migration rate (*p* < 0.0001) of equine skeletal muscle cells, whereas knockdown of *IGF1* significantly inhibited these cellular capabilities. Mechanistic studies revealed that *IGF1* overexpression significantly increased Akt phosphorylation. Notably, *IGF1* exerted its biological functions in conjunction with the activation of the PI3K/Akt signaling pathway. In conclusion, *IGF1* enhances cell proliferation and wound-healing capacity of primary equine skeletal muscle cells, which correlates with its ability to activate the PI3K/Akt signaling pathway. This study is the first to reveal the critical role of *IGF1* in equine skeletal muscle biology at the cellular level, providing experimental evidence for a deeper understanding of the molecular mechanisms underlying the formation of athletic performance in *Yili horses*. It also offers novel potential targets for the molecular breeding of sport horses and interventions for injury repair.

## Simple Summary

The athletic performance of *Yili horses*, particularly their speed and endurance, is closely linked to the biological functions of skeletal muscle. While the *IGF1* gene has been identified as a key player in exercise response, its specific role in equine skeletal muscle cells remains unclear. In this study, we manipulated *IGF1* expression in primary equine skeletal muscle cells using overexpression and RNA interference (RNAi) techniques. We found that elevated *IGF1* levels significantly enhanced cell proliferation and migration, whereas *IGF1* knockdown suppressed these abilities. Mechanistically, these effects were associated with the activation of the PI3K/Akt signaling pathway, as evidenced by increased Akt phosphorylation. Our findings demonstrate that the *IGF1*/PI3K/Akt axis positively regulates the phenotype of horse skeletal muscle cells. This study provides a cellular-level basis for understanding muscle adaptation in sport horses and suggests potential molecular targets for breeding and injury recovery.

## 1. Introduction

The Yili horse, a distinguished cultivated breed renowned for its exceptional endurance, speed, and adaptability to high-altitude environments, represents a versatile and superior equine breed [1]. With the increasing demand for modern equestrian sports and leisure riding, enhancing the exercise performance of *Yili horses* has become a critical breeding objective. Exercise performance is a complex biological trait regulated by multiple genes and pathways, with its physiological foundation primarily involving the coordinated function of the skeletal muscle, cardiovascular, respiratory, and nervous systems [2,3]. As the primary effector organ of movement, skeletal muscle—specifically its contractile capacity, energy metabolism, and damage repair/regenerative abilities—directly determines a horse’s speed, stamina, and power [4,5]. Through integrated transcriptomic and metabolomic analyses, Wang [6] demonstrated that differentially expressed genes and metabolites in *Yili horses* before and after an 80 km endurance race are primarily associated with regulatory mechanisms involving energy metabolism, oxidative stress, and inflammatory responses. Similarly, Huang [7] identified genes related to cardiac adaptive remodeling and energy supply, such as *CCL3*, *CSF1*, *CSF3*, and *IFNG*, by analyzing differential gene expression in blood and correlating it with cardiac parameters following a 5000 m race. Furthermore, whole-transcriptome analysis of blood samples from *Yili horses* before and after a 5000 m race [8] revealed significant upregulation of *IGF1*, which is involved in the PI3K-Akt signaling pathway. Collectively, these studies not only highlight the potential of whole-transcriptome technologies in equine exercise performance research but also provide a valuable candidate gene pool for subsequent functional validation and mechanistic exploration. Insulin-like Growth Factor 1 (*IGF1*) is a multifunctional peptide hormone primarily synthesized in the liver, regulating cellular functions via paracrine or autocrine mechanisms [9,10]. Studies indicate that *IGF1* enhances protein synthesis rates within myocytes while inhibiting protein degradation through the activation of downstream signaling pathways, thereby promoting muscle fiber hypertrophy [11]. Recent research has also demonstrated that *IGF1* induces directional migration in various cell types, including myoblasts [12]; in vitro experiments have confirmed that *IGF1* significantly enhances the migratory capacity of C2C12 myoblasts [13]. Although specific studies on *IGF1*-mediated migration in equine muscle cells remain limited, its established roles in other species suggest that it may serve as a crucial regulatory factor in equine skeletal muscle repair.

Among the myriad biological effects mediated by *IGF1*, the PI3K/Akt signaling pathway serves as a pivotal signaling hub, playing crucial roles in the regulation of cell survival, proliferation, growth, and metabolism [14]. Activation of this pathway is initiated by the binding of *IGF1* to its receptor, *IGF1R*, on the cell membrane. Activated *IGF1R* further phosphorylates intracellular substrate proteins, most notably members of the insulin receptor substrate (*IRS*) family, such as *IRS-1* [15]. Phosphorylated *IRS-1* activates class I PI3K, which catalyzes the conversion of phosphatidylinositol 4,5-bisphosphate (*PIP2*) to phosphatidylinositol (3,4,5)-trisphosphate (*PIP3*) on the cell membrane. Acting as a second messenger, *PIP3* translocates the signal from the membrane to the cytoplasm, where Akt is fully activated via phosphorylation by kinases such as phosphoinositide-dependent kinase 1 (*PDK1*) [16,17]. The PI3K/Akt pathway plays a significant role in regulating cell migration by influencing cytoskeletal reorganization [18]. It has been confirmed that *IGF1* enhances the proliferation and migration capacities of bone marrow mesenchymal stem cells (BMSCs), specifically through the activation of the PI3K/Akt signaling pathway [19].

In the skeletal muscle of *Yili horses*, *IGF1*—a core gene upregulated in response to exercise stress—is hypothesized to promote the proliferation and migration capabilities of equine primary skeletal muscle cells via activation of the canonical PI3K/Akt signaling pathway. To test this scientific hypothesis, this study aims to utilize equine primary skeletal muscle cells as a model. Through *IGF1* overexpression and knockdown experiments, we seek to elucidate the activation effect of *IGF1* on the PI3K/Akt signaling cascade and its regulatory role in cell proliferation and migration. This research aims to uncover the key molecular mechanisms underlying the superior exercise performance of *Yili horses*, providing theoretical foundations and potential molecular targets for the selective breeding of athletic horses.

## 2. Materials and Methods

### 2.1. Cell Culture of Equine Primary Skeletal Muscle Cells

Skeletal muscle tissue was obtained from the gluteus medius muscle of one healthy adult Yili horse (male, 4 years old) sourced from the Xinjiang Changji Tengma Stud Farm, with collection performed under strict aseptic conditions. Notably, all primary cells utilized in this study were derived from this single donor (*n* = 1). All animal procedures were approved by the Institutional Animal Care and Use Committee of Animal Welfare and Ethics Committee of Xinjiang Agricultural University (Approval No. 2024003). The collected tissues were immediately placed in pre-cooled phosphate-buffered saline (PBS; Gibco, Grand Island, NY, USA) containing 2% penicillin-streptomycin (Solarbio, Beijing, China) and transported to the laboratory on ice within 2 h.

For primary cell isolation, the tissues were washed three times with PBS to remove blood and connective tissue. Subsequently, the tissues were minced into 1–2 mm^3^ fragments and digested with 0.25% trypsin-EDTA at 37 °C for 30 min, followed by a second digestion with 0.1% collagenase type II at 37 °C for 4–6 h. The dissociated cells were filtered through a 70 μm cell strainer, centrifuged at 1000 rpm for 5 min, and resuspended in growth medium consisting of Dulbecco’s Modified Eagle Medium/Nutrient Mixture F-12 supplemented with 20% fetal bovine serum and 1% penicillin-streptomycin. The cells were then seeded into 25 cm^2^ culture flasks and incubated at 37 °C in a humidified atmosphere containing 5% CO_2_. The culture medium was replaced every 48 h. Upon reaching 80% confluence, cells were subcultured using 0.25% trypsin-EDTA for subsequent experiments. Only cells at passages 2 to 4 (P2–P4) were used for all functional assays to ensure stable phenotypes and minimize spontaneous differentiation.

### 2.2. Immunofluorescence Staining of Myogenic Markers

Primary equine skeletal muscle cells at passage 2 were seeded onto sterile glass coverslips placed in 12-well plates at a density of 1 × 10^5^ cells/well and cultured in a humidified incubator at 37 °C with 5% CO_2_ until they reached 70–80% confluence. For immunofluorescence staining, cells were washed twice with pre-warmed phosphate-buffered saline (PBS; Servicebio, Wuhan, Hubei, China), followed by fixation with 4% paraformaldehyde (PFA; Servicebio, Wuhan, Hubei, China) for 15 min at room temperature. After fixation, cells were permeabilized with 0.5% Triton X-100 in PBS for 10 min and subsequently blocked with 5% bovine serum albumin (BSA) in PBS for 1 h at room temperature to minimize non-specific binding. Cells were then incubated overnight at 4 °C with the following primary antibodies diluted in 5% BSA: rabbit anti-Pax7 polyclonal antibody (1:200, Proteintech, Wuhan, Hubei, China) and mouse anti-Desmin recombinant monoclonal antibody (1:300, Proteintech, Wuhan, Hubei, China). Following three washes with PBS, cells were incubated with corresponding secondary antibodies for 1 h at room temperature in the dark: Alexa Fluor 488-conjugated Goat Anti-Rabbit IgG (H + L) and Alexa Fluor 594-conjugated Goat Anti-Mouse IgG (H + L), both diluted 1:500 (Thermo Fisher Scientific, Waltham, MA, USA). Nuclear counterstaining was performed using 4′,6-diamidino-2-phenylindole (DAPI; 1 µg/mL) for 5 min at room temperature. Coverslips were then mounted onto glass slides and the cells were visualized using an NIB610-FL inverted fluorescence microscope (Ningbo Yongxin Optics Co., Ltd., Ningbo, Zhejiang, China).

### 2.3. Plasmid Transfection

Equine primary skeletal muscle cells in the logarithmic growth phase were seeded into 6-well plates at a density of 5 × 10^5^ cells per well and cultured overnight in a 37 °C incubator with 5% CO_2_ to allow for attachment. Two hours prior to transfection, the original medium was removed and replaced with serum-free MEM medium. The transfection complexes were prepared at room temperature. Briefly, 4 μg of plasmid DNA or 10 μL of siRNA (20 μM) was diluted in 100 μL of serum-free MEM medium, gently mixed, and incubated for 5 min. Simultaneously, 5 μL of Chemi-Trans™ Lipofectin Transfection Reagent (Jingkede Biotechnology (Wuhan) Co., Ltd., Wuhan, Hubei, China) was diluted in 100 μL of serum-free MEM medium and incubated for 5 min. The two dilutions were then gently combined (total volume: 200 μL) and allowed to stand for 15 min to form DNA/RNA-liposome complexes. The 200 μL of complexes was added dropwise to the corresponding wells, and the plate was gently rocked back and forth to ensure even distribution. After 6 h of incubation, the transfection medium was aspirated and replaced with fresh complete growth medium. Cells were further cultured for a total of 48 h post-transfection prior to subsequent assays.

### 2.4. RT-qPCR

Total RNA was extracted from cells using an Animal Cell Total RNA Extraction Kit, and RNA concentration and purity were assessed using a micro-spectrophotometer (A260/A280 ratio). Subsequently, 1 μg of total RNA was reverse transcribed into cDNA using the SynScript^®^ III RT SuperMix for qPCR kit (Tsingke Biotechnology Co., Ltd. Beijing, China). Quantitative PCR was performed on an ABI QuantStudio StepOne Plus system using ArtiCanCE™ SYBR qPCR Mix (Thermo Fisher, Waltham, MA, USA). The 20 μL reaction system consisted of 10 μL of SYBR Green Mix (Thermo Fisher, Waltham, MA, USA), 0.8 μL of forward and reverse primers (10 μM each), 1 μL of cDNA template, and 7.4 μL of RNase-free water. The thermal cycling protocol was as follows: initial denaturation at 95 °C for 5 min, followed by 40 cycles of 95 °C for 15 s, 60 °C for 20 s, and 72 °C for 20 s. *GAPDH* was employed as the internal reference gene, and the relative expression level of *IGF1* was calculated using the 2^−ΔΔCt^ method. Primer sequences are listed in Table S1.

### 2.5. Western Blot

Cells were lysed on ice for 30 min using RIPA lysis buffer (Thermo Fisher, Waltham, MA, USA) supplemented with a protease inhibitor cocktail. The lysates were subsequently centrifuged at 12,000 rpm for 10 min at 4 °C, and the supernatants were collected. Protein concentrations were determined using a BCA assay kit. Equal amounts of protein samples (30 μg per lane) were mixed with 5× loading buffer and boiled for 10 min to denature the proteins. The denatured proteins were separated via 10% SDS-PAGE and transferred onto polyvinylidene fluoride (PVDF) membranes (0.45 μm pore size) using a wet transfer method (300 mA, 30 min). The membranes were blocked with Tris-buffered saline with Tween-20 (TBST) containing 3% bovine serum albumin (BSA) at room temperature for 30 min. After blocking, the membranes were incubated overnight at 4 °C with the following specific primary antibodies: anti-GAPDH (Proteintech, Wuhan, Hubei, China 1:10,000 dilution); anti-Akt (Invitrogen, Thermo Fisher Scientific, Waltham, MA, USA, 1:1000 dilution); and anti-p-Akt (Ser473) (Invitrogen, Thermo Fisher Scientific, Waltham, MA, USA 1:1000 dilution). Following three washes with TBST, the membranes were incubated with corresponding HRP-conjugated secondary antibodies (1:5000 dilution) at room temperature for 30 min. Protein signals were visualized using an ultra-sensitive ECL chemiluminescence kit and a chemiluminescence imaging system. The grayscale values of the target bands were semi-quantitatively analyzed using AlphaEaseFC software (version4.0, Alpha Innotech, San Leandro, CA, USA). Data were normalized to GAPDH, and the relative protein expression levels were calculated.

### 2.6. Cell Proliferation Assay

Equine primary skeletal muscle cells were seeded into 96-well plates at a density of 5 × 10^3^ cells per well and cultured until attachment. Following the grouping strategy outlined in Section 2.2, cells were subjected to the respective treatments. To assess proliferation, 10 μL of CCK-8 solution was added to each well at the indicated time points (0, 24, and 48 h post-treatment). The plates were returned to the incubator for an additional 1–2 h. Finally, the absorbance (optical density, OD) at 450 nm was measured using a microplate reader to evaluate cell viability. Each assay was performed in triplicate.

### 2.7. Scratch Wound-Healing Assay

Equine primary skeletal muscle cells in the logarithmic growth phase were seeded into 6-well plates at a density of 5 × 10^5^ cells per well and cultured until they reached approximately 90% confluence. A sterile 200 μL pipette tip was used to create a linear scratch perpendicular to the plate bottom. Cellular debris was removed by gently washing three times with phosphate-buffered saline (PBS). Subsequently, the medium was replaced with serum-free medium. Baseline images of the scratch (0 h) were immediately captured using an inverted microscope. The cells were then returned to the incubator for continued culture. Images were acquired again at the same positions at 24 h and 48 h post-scratch. The scratch area was quantitatively measured using ImageJ software (version 1.53k; National Institutes of Health, Bethesda, MD, USA).

### 2.8. Statistical Analysis

All experiments were independently repeated at least three times. Data are presented as the mean ± standard deviation (SD). Statistical analyses were performed using GraphPad Prism 8.0 (GraphPad Software, LLC, Boston, MA, USA). Comparisons between two groups were assessed using Student’s *t*-test. For comparisons among multiple groups, one-way analysis of variance (ANOVA) was applied, followed by Tukey’s post-hoc test for pairwise comparisons. A *p*-value < 0.05 was considered statistically significant.

## 3. Results and Analysis

### 3.1. Characterization of Primary Equine Skeletal Muscle Cells

**Figure 1:**
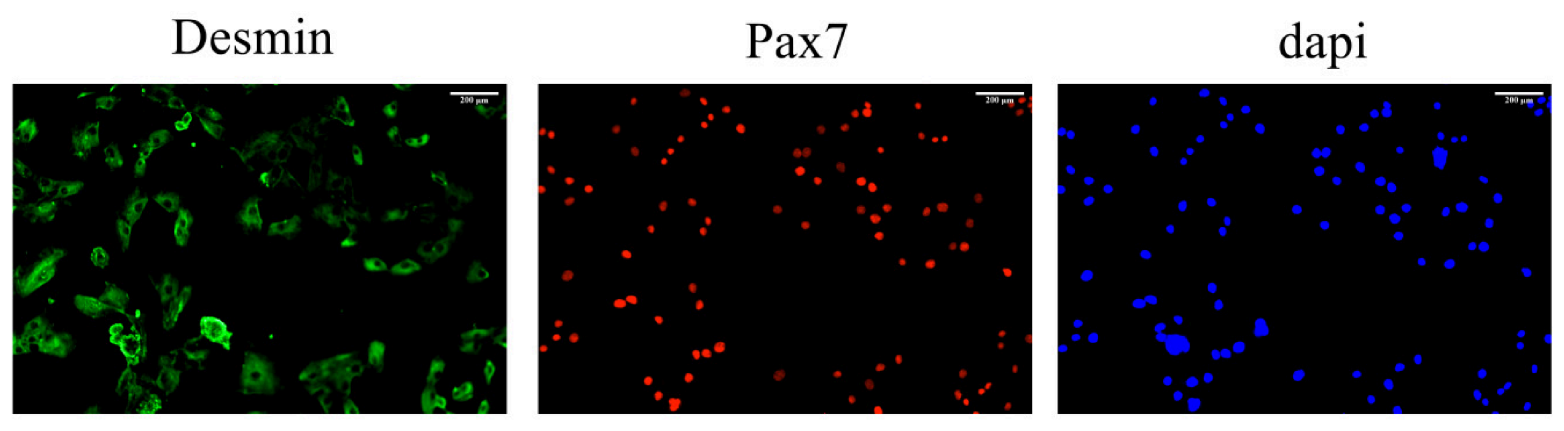
Immunofluorescence characterization of primary equine skeletal muscle cells.

To confirm the myogenic identity of the cultured cells, immunofluorescence staining for Pax7 (satellite cell marker) and Desmin (muscle-specific cytoskeletal marker) was performed. As shown in Figure 1, the majority of cells exhibited strong cytoplasmic Desmin staining (green) and nuclear Pax7 staining (red), with both signals co-localizing with DAPI-labeled nuclei (blue).

### 3.2. Construction and Validation of the IGF1 Overexpression Vector

**Figure 2:**
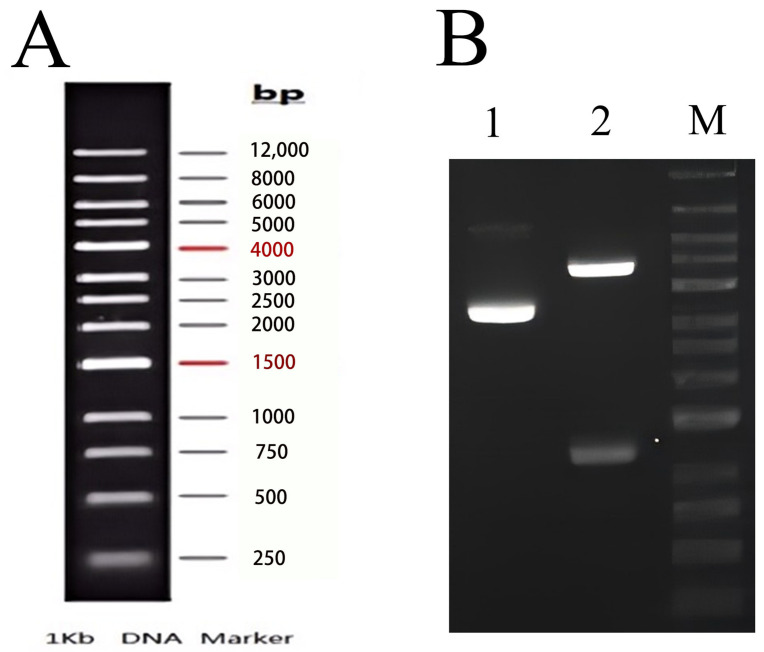
Validation of the pcDNA3.1-*IGF1* overexpression vector. (**A**) The 1 Kb DNA Marker used for size estimation in agarose gel electrophoresis. The red lines indicate the 4000 bp and 1500 bp markers. (**B**) Agarose gel electrophoresis of the recombinant plasmid. Lane M: DNA Marker; Lane 1: empty pcDNA3.1 plasmid; Lane 2: plasmid digested with MluI and XhoI.

To ensure the reliability of subsequent functional experiments, the constructed pcDNA3.1-*IGF1* overexpression plasmid was rigorously validated at the molecular level. First, double digestion verification was performed using the restriction endonucleases MluI and XhoI. As shown in Figure 2, agarose gel electrophoresis revealed distinct bands corresponding to the expected sizes of both the inserted *IGF1* fragment and the linearized vector backbone, preliminarily confirming the correctness of the plasmid construction.

Subsequently, Sanger sequencing was conducted to analyze the insert sequence (Figure S1). Alignment of the sequencing results with the equine *IGF1* reference sequence (GenBank Accession No. NC_091711.1) demonstrated 100% sequence identity, with no frameshift mutations or unexpected base substitutions detected. These results collectively indicate the successful construction of the recombinant plasmid for the overexpression of equine *IGF1*.

### 3.3. RT-qPCR Results and Validation

**Figure 3:**
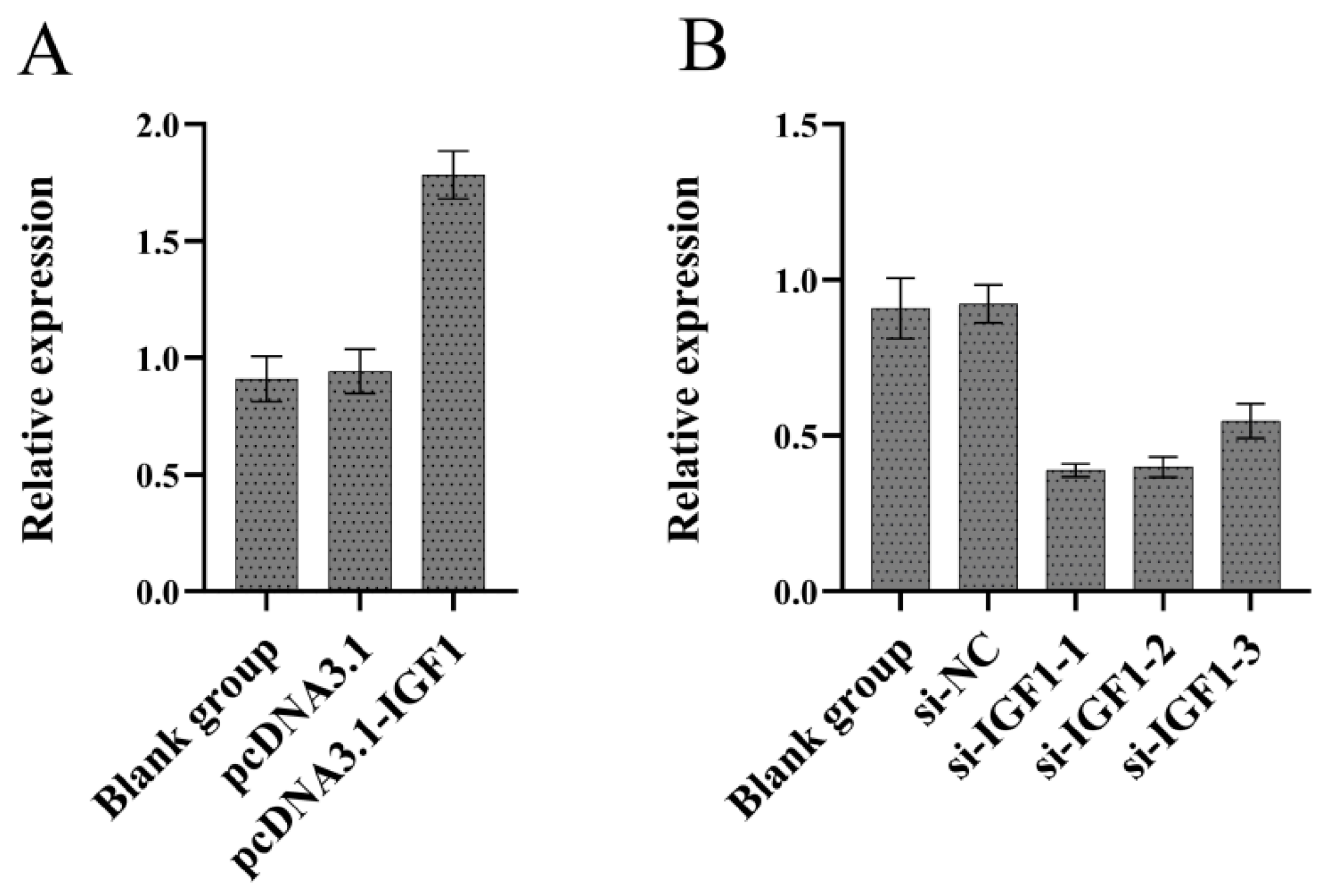
Validation of *IGF1* overexpression and knockdown efficiency via RT-qPCR. (**A**) Relative *IGF1* expression in cells transfected with the pcDNA3.1-*IGF1* overexpression vector (OE-*IGF1*) compared to the empty vector control (OE-NC). (**B**) Knockdown efficiency of three different siRNA constructs (si-*IGF1*-1, si-*IGF1*-2, si-*IGF1*-3) compared to the negative control (si-NC).

RT-qPCR analysis was performed to validate the transfection efficiency of the constructed vectors (Figure 3). In the overexpression assay, the relative *IGF1* level in the pcDNA3.1-*IGF1* group was significantly higher than that in the empty vector control (OE-NC) and the Blank group (BG) (*p* < 0.01), indicating that the recombinant plasmid was successfully expressed in equine skeletal muscle cells and was suitable for subsequent functional assays.

Regarding the knockdown assay, all three siRNA constructs (si-*IGF1*-1, si-*IGF1*-2, and si-*IGF1*-3) significantly reduced *IGF1* expression compared to the negative control (si-NC) and BG (*p* < 0.01). Among them, si-*IGF1*-1 exhibited the most potent silencing effect. Therefore, si-*IGF1*-1 was selected for all subsequent interference experiments.

### 3.4. IGF1 Promotes the Proliferation and Migration of Equine Skeletal Muscle Cells

**Figure 4:**
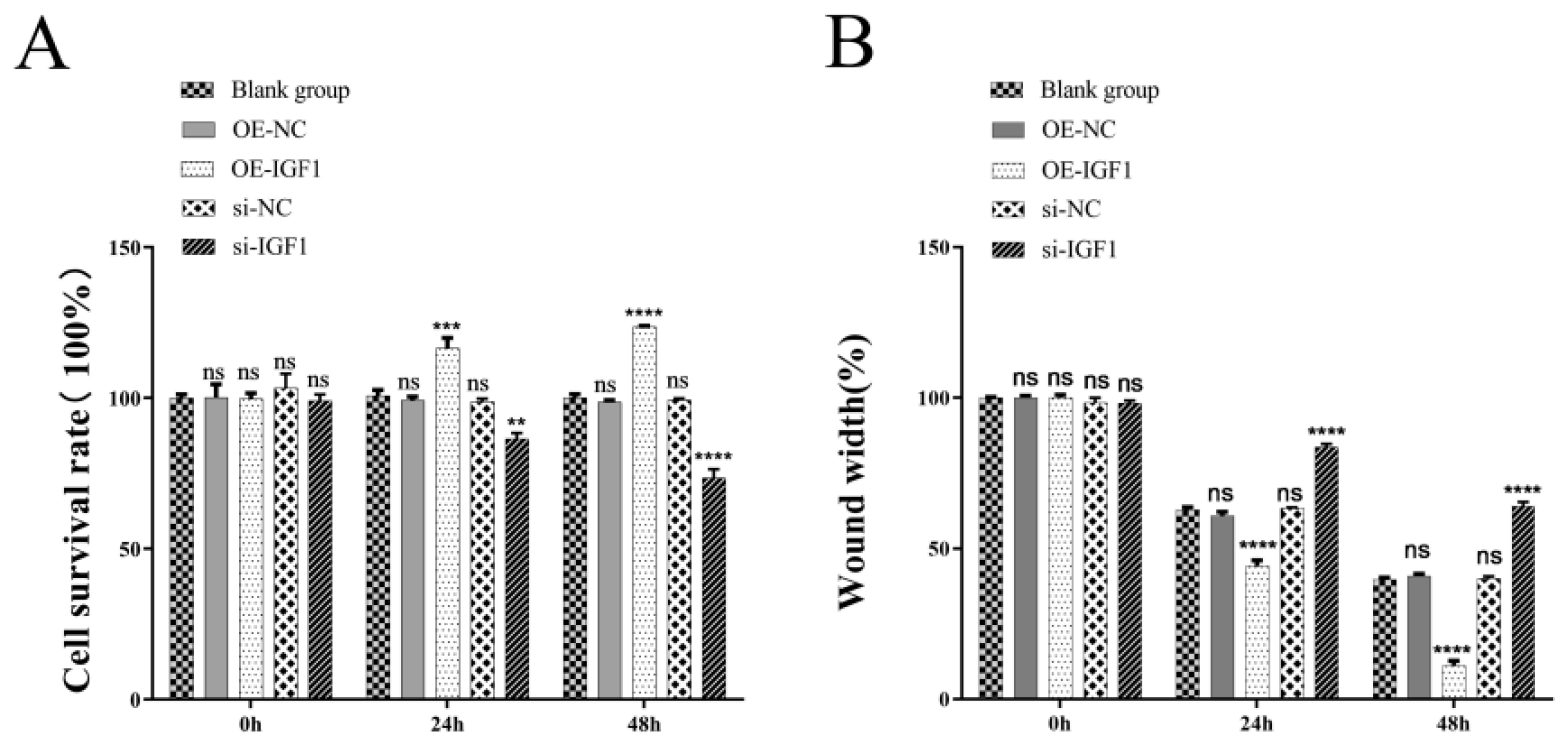
Effects of *IGF1* overexpression and knockdown on equine skeletal muscle cells. (**A**) CCK-8 assays measuring cell viability of equine skeletal muscle cells. (**B**) Effect of *IGF1* overexpression and knockdown on cell migration capacity (assessed by scratch wound-healing assay). Note: ns: not significant; **: *p* = 0.0013; ***: *p* = 0.004; ****: *p* < 0.0001.

CCK-8 assays demonstrated that *IGF1* expression significantly affected the viability of equine skeletal muscle cells (Figure 4A). At 0 h post-transfection, no significant differences in cell viability were observed among the groups. However, at 24 h, the viability of the OE-*IGF1* group was significantly higher than that of the OE-NC group (*p* = 0.004), while the si-*IGF1* group exhibited a marked decrease compared to the si-NC group (*p* = 0.0013). By 48 h, cell viability in the OE-*IGF1* group was drastically elevated compared to the OE-NC group (*p* < 0.0001), whereas the si-*IGF1* group showed a drastic reduction compared to the si-NC group (*p* < 0.0001). These results collectively indicate that *IGF1* overexpression significantly promotes cell proliferation, whereas *IGF1* knockdown markedly inhibits it.

**Figure 5:**
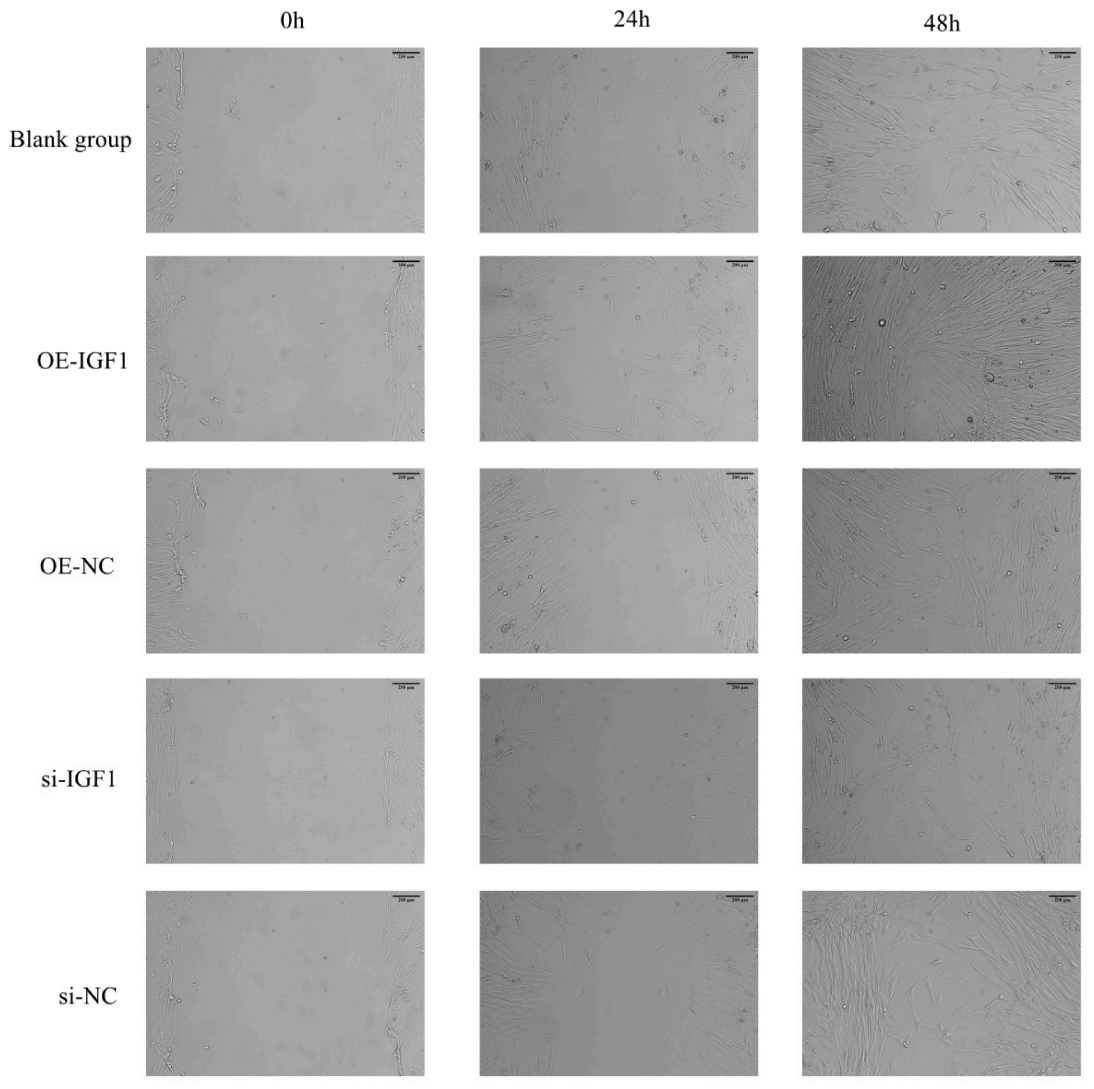
Representative microscopic images of the scratch wound-healing assay at 0, 24, and 48 h post-scratch. Each scale bar = 200 μm.

Similarly, scratch wound-healing assays revealed that *IGF1* expression profoundly regulated cell migration capacity (Figure 4B and Figure 5). At 0 h post-scratch, the initial wound widths were consistent across all groups. At 24 h, the wound width percentage in the OE-*IGF1* group was significantly lower than that in the OE-NC group (*p* < 0.0001), while the si-*IGF1* group displayed a significantly higher percentage compared to the si-NC group (*p* < 0.0001). By 48 h, the OE-*IGF1* group exhibited the fastest wound closure rate, with a wound width percentage drastically lower than that of the OE-NC group (*p* < 0.0001). Conversely, the si-*IGF1* group maintained a drastically higher wound width percentage than the si-NC group (*p* < 0.0001). These findings indicate that *IGF1* modulates the wound-healing capacity of equine skeletal muscle cells, likely reflecting a combination of enhanced cell motility and increased proliferation.

### 3.5. Effects of IGF1 Overexpression and Knockdown on the PI3K/Akt Signaling Pathway

**Figure 6:**
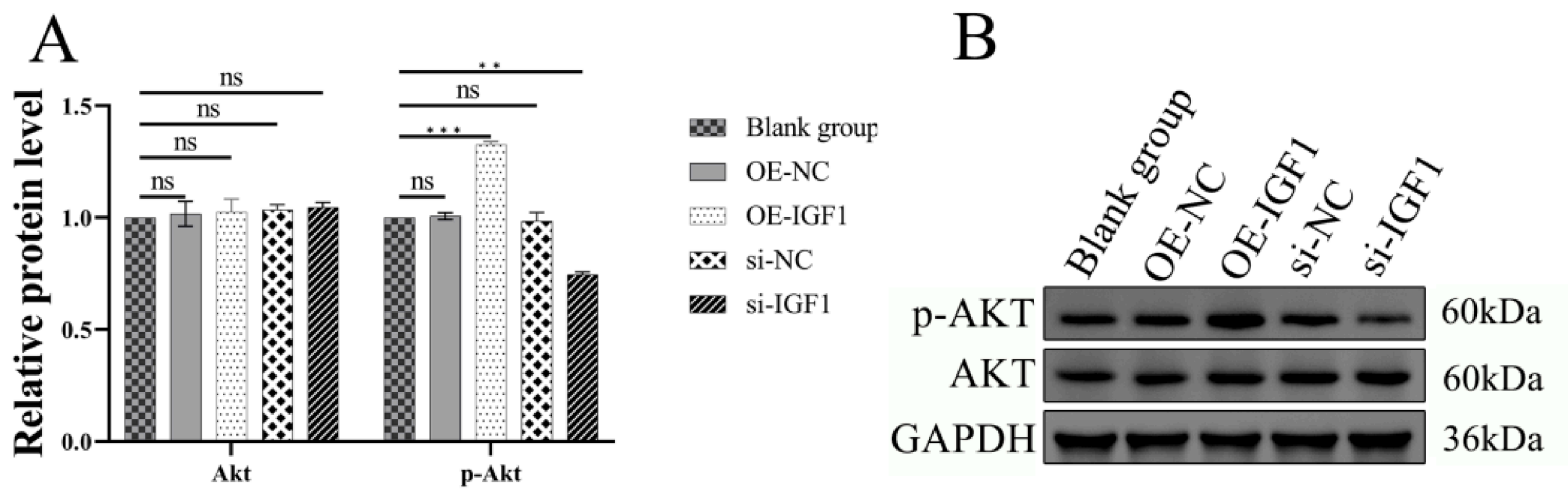
*IGF1* activates the PI3K/Akt signaling pathway in equine skeletal muscle cells. (**A**) Quantitative analysis of relative protein expression levels via band intensity; (**B**) representative Western blot bands showing phosphorylated Akt (p-Akt), total Akt, and GAPDH (loading control). Note: ns: not significant; **: *p* = 0.0029; ***: *p* = 0.0007.

Western blot analysis was performed to evaluate the effects of *IGF1* overexpression and knockdown on Akt phosphorylation, a canonical activation marker of the PI3K/Akt signaling cascade (Figure 6B). Full uncropped blots for all target proteins, along with raw data from three independent biological replicates, are provided in File S1 to ensure experimental transparency. Quantitative analysis of band intensity, conducted using AlphaEase FC software (version4.0, Alpha Innotech, San Leandro, CA, USA), revealed that *IGF1* modulated the phosphorylation status of Akt without altering total protein levels (Figure 6A). Specifically, compared to the control groups, the relative protein expression level of phosphorylated Akt (p-Akt) was significantly elevated in the OE-*IGF1* group, whereas it was markedly reduced in the si-*IGF1* group. Notably, the p-Akt levels in the negative control groups (OE-NC and si-NC) were not significantly different compared to the control. In contrast, the relative expression levels of total Akt remained stable across all groups, fluctuating slightly around 1.0 with no statistically significant differences. These results demonstrate that *IGF1* regulates Akt phosphorylation in equine skeletal muscle cells. While this observation is consistent with the activation of the PI3K/Akt signaling pathway, it is based solely on downstream Akt activation status without direct assessment of upstream PI3K activity or pathway inhibition.

## 4. Discussion

The growth, development, and homeostasis of skeletal muscle tissue are fundamentally dependent on the basic biological process of cell proliferation [20]. Among these processes, skeletal muscle satellite cells (SCs), as the major population of muscle stem cells in adult skeletal muscle, play a decisive role in determining the replenishment of myofiber numbers, adaptive hypertrophy, and regenerative efficiency post-injury through their activation, proliferation, and directional migration capacities [21,22]. Unlike standard laboratory rodents, horses represent a unique physiological model due to their massive body size, high aerobic capacity, and predisposition to exercise-induced muscle damage [23]. Rodent myoblasts typically exhibit rapid proliferation and differentiation kinetics that differ markedly from those of large mammals [24]. By focusing on equine cells, our data provide a more accurate representation of the cellular milieu relevant to equine sports medicine. Following high-intensity exercise-induced damage, dynamic changes in various growth factors within the local microenvironment act as key signals initiating repair programs [25]. The PI3K/Akt pathway represents one such regulatory axis, with its signaling tightly controlled by multiple mechanisms to elicit specific cellular responses [26]. Insulin-like Growth Factor 1 (*IGF1*), a 70-amino acid polypeptide containing three disulfide bonds and belonging to the insulin family [27], has been shown to promote cell cycle progression in numerous normal and cancer cell types by activating the PI3K/Akt signaling cascade and regulating cyclin-dependent kinases [28]. While studies have explored inhibiting *IGF1* function to slow tumor growth by affecting cell proliferation, survival, and migration/invasion [29], *IGF1* is also recognized as a core paracrine/autocrine factor governing skeletal muscle satellite cell behavior. Its expression is significantly upregulated following exercise and positively correlates with muscle recovery rates [30].

In this study, we performed *IGF1* overexpression and knockdown in equine primary skeletal muscle cells. CCK-8 and cell scratch assays demonstrated that *IGF1* overexpression significantly enhanced the proliferation and migration capacities of equine skeletal muscle cells, whereas *IGF1* knockdown exerted a marked inhibitory effect. This aligns with findings by McDonald et al., who reported improved embryo survival rates 30 days post-transfer after supplementing bovine embryos with exogenous *IGF1* [31]. Conversely, Cong Dong et al. [32] revealed the complex functionality of *IGF1*, showing that blocking its expression attenuated type 2 inflammatory responses, mucin production, and goblet cell hyperplasia, highlighting its potential pro-proliferative role in pathological inflammation and tissue remodeling. Greisha [33] identified molecular interactions between *CYR61* and *IGF1*, demonstrating that PI3K/Akt signaling mediates *IGF1*-induced *CYR61* expression, thereby promoting the survival, migration, proliferation, metabolism, and self-renewal of stem-like cell populations. Similarly, Yuan et al. [34] showed that miR-145-5p inhibits proliferation and differentiation in porcine skeletal muscle satellite cells by targeting *IGF1*, thereby suppressing the Akt pathway—a finding reciprocal to our gain-of-function results via pathway activation.

To elucidate the molecular mechanisms underlying *IGF1*-regulated cellular functions, we assessed the activity changes in its downstream key PI3K/Akt signaling pathway. Western blot analysis further revealed that *IGF1* stimulation significantly increased Akt phosphorylation levels without altering total Akt protein expression. This indicates that *IGF1* does not regulate Akt expression but specifically activates its kinase activity through post-translational phosphorylation modification [35], consistent with the established characteristics of the *IGF1*-PI3K-Akt signaling axis observed in fibroblasts, neuronal precursor cells, and various tumor cell lines [36,37]. As a core effector molecule downstream of PI3K, activated p-Akt may coordinately regulate cellular functions through multiple pathways [38]. Specifically, p-Akt can directly phosphorylate glycogen synthase kinase 3β (GSK3β) and FoxO family transcription factors (FoxO1/FoxO3a) [39], leading to their cytoplasmic retention and transcriptional inactivation. This relieves GSK3β-mediated ubiquitin-dependent degradation of Cyclin D1 and FoxO-mediated transcriptional activation of pro-apoptotic genes (e.g., Bim, FasL) and cell cycle inhibitors (e.g., p27Kip1). Consequently, this promotes the formation of Cyclin D1/CDK4 complexes, Rb protein phosphorylation, G1/S phase transition, and upregulates anti-apoptotic proteins like Bcl-2 and Mcl-1, significantly enhancing cellular resistance to apoptosis induced by serum deprivation or oxidative stress [40,41,42]. Furthermore, p-Akt activates the mTORC1 complex, enhancing ribosome biogenesis and translation initiation efficiency, thereby providing the material foundation for rapid cell proliferation [43]. In summary, *IGF1* promotes the proliferation and migration of equine primary skeletal muscle cells by activating the PI3K/Akt signaling pathway and potentially its downstream regulatory network. It is important to note that while our study demonstrates a clear correlation between *IGF1* expression and Akt phosphorylation, and aligns with the established *IGF1*-PI3K/Akt signaling axis in other cell types, we did not employ pharmacological inhibitors (e.g., LY294002 or Wortmannin) or genetic silencing of PI3K to confirm the necessity of this pathway in mediating the observed phenotypic changes. Therefore, we cannot exclude the possibility that other parallel signaling pathways might contribute to the *IGF1*-induced proliferation and migration of equine skeletal muscle cells.

Several limitations of the present study warrant consideration. First, regarding the scratch wound-healing assay, the absence of pretreatment with a proliferation inhibitor (e.g., mitomycin-C) means that the observed wound closure reflects both cell migration and *IGF1*-driven proliferation. Second, the primary skeletal muscle cells were isolated from only one donor horse; thus, we were unable to account for inter-individual biological variability, and our findings reflect the biological characteristics of this specific individual. Third, while we observed increased phosphorylation of Akt, our mechanistic investigation did not extend to measuring upstream PI3K activity or employing specific PI3K inhibitors. Consequently, although our data suggest the involvement of the PI3K/Akt signaling cascade, we cannot definitively conclude that this is the predominant or exclusive pathway responsible for the observed phenotypes, as other signaling axes may also be activated by *IGF1*. Future studies utilizing time-lapse microscopy, transwell assays with proliferation blockers, and pharmacological inhibition of the PI3K/Akt pathway—conducted on cells from multiple donors—are imperative to decouple these cellular processes and validate the specific contributions of this signaling axis. Additionally, future work should incorporate physiological *IGF1* stimulation or exercise-mimetic interventions (e.g., cyclic mechanical stretch) to better recapitulate in vivo regulatory contexts, and extend these investigations to in vivo equine models to verify the role of *IGF1* in skeletal muscle repair and exercise adaptation.

Despite the aforementioned limitations, these findings offer valuable insights into the molecular mechanisms governing skeletal muscle physiology in horses. Using an equine primary skeletal muscle cell model, we observed that *IGF1* overexpression was closely associated with enhanced cell proliferation and wound-healing capacity, concomitant with upregulated phosphorylation of Akt, a key effector in the PI3K/Akt signaling pathway. These results suggest that *IGF1* may play a role in regulating the cellular response of equine skeletal muscle to physiological stress, providing a novel entry point for deciphering the molecular networks underlying equine athletic performance. It is important to note, however, that the current data do not permit definitive conclusions regarding whether the PI3K/Akt axis acts as the sole regulatory hub for the observed phenotypes. Further validation using multi-donor samples and in vivo models is required to substantiate the feasibility of targeting this axis for selective breeding or performance interventions in sport horses.

## 5. Conclusions

This study successfully established *IGF1* overexpression and knockdown models in equine primary skeletal muscle cells derived from a single donor. Functional assays demonstrated that *IGF1* overexpression significantly promoted cell proliferation and enhanced wound-healing capacity, whereas *IGF1* knockdown exerted a marked inhibitory effect. Mechanistically, these phenotypic changes coincided with increased Akt phosphorylation, suggesting an association with the PI3K/Akt signaling axis.

## Data Availability

The results of the transcriptomic sequencing of horse blood have been deposited in the BioProject under accession number PRJNA1250149.

## Supplementary Materials

The following supporting information can be downloaded at: https://www.mdpi.com/article/10.3390/biology15151225/s1, Table S1: Primers used in this study; Figure S1: Sequencing chromatogram of the 5′-end of the *IGF1* insert cloned into pEGFP-N; File S1: Full uncropped blots for all target proteins.
